# The Best of Both Worlds: The Role of Career Adaptability and Career Competencies in Students’ Well-Being and Performance

**DOI:** 10.3389/fpsyg.2018.01678

**Published:** 2018-09-12

**Authors:** Jos Akkermans, Kristina Paradniké, Beatrice I. J. M. Van der Heijden, Ans De Vos

**Affiliations:** ^1^School of Business and Economics, Vrije Universiteit Amsterdam, Amsterdam, Netherlands; ^2^Institute of Psychology, Mykolas Romeris University, Vilnius, Lithuania; ^3^Institute for Management Research, Radboud University, Nijmegen, Netherlands; ^4^Faculty of Management, Science, and Technology, Open University of the Netherlands, Heerlen, Netherlands; ^5^Kingston Business School, Kingston University, London, United Kingdom; ^6^Business School, Hubei University, Wuhan, China; ^7^Department of Management, University of Antwerp, Antwerp, Belgium; ^8^Antwerp Management School, Antwerp, Belgium

**Keywords:** career adaptability, career competencies, academic major satisfaction, study engagement, life satisfaction, academic performance, job demands-resources theory

## Abstract

In addition to acquiring occupation-specific knowledge and skills, students need to develop a set of career self-management skills – or resources – that helps them to successfully maneuver the various career-related challenges they face and that stimulate their well-being, engagement, and performance in studying tasks. In the current study, we apply the Job Demands-Resources (JD-R) theory in an educational setting and suggest that career adaptability and career competencies are important career resources that predict both life satisfaction and academic performance via students’ satisfaction with the choice of their major and study engagement. Undergraduate students (*N* = 672) from nine different colleges and universities in Lithuania participated in the study. The results revealed that career adaptability and career competencies were positively linked to students’ life satisfaction, both directly and via study engagement. In addition, these career resources were positively, yet indirectly, related to academic performance via study engagement. Overall, the results suggest that career resources contribute to study engagement, life satisfaction, and academic performance. The results of our study further support JD-R theorizing and its applicability in student samples. Further theoretical and practical implications are discussed.

## Introduction

Recent labor market developments – such as globalization, rapid technological advancements, and the increasing use of outsourcing, part-time and temporary employees – have significantly altered the work context, creating changes in how individuals manage their career (Sullivan and Baruch, 2009). Furthermore, these changes also have a fundamental effect on students’ career development (Schoon et al., 2001). The transition from higher education to work has always been a stressful period for young adults, with students suffering from stress brought along by such factors as examinations, study tasks, leaving home, and financial pressures (Robotham and Julian, 2006). Yet, today’s labor market also requires students to already start planning and managing their long-term careers during their studies. For example, over and above good performance throughout their curriculum, students need to develop work readiness and employability skills, explore possible career paths, form more specific vocational goals and plans, and act to implement those goals (Lent and Brown, 2013). Taken together, it is clear that young adults have to start proactively managing their careers already during their studies if they are to make a successful transition into the labor market (Koen et al., 2012). In order to achieve this, they need certain resources and competencies that help them to successfully manage their (study) career and to stimulate their well-being and performance (Hirschi, 2012).

Thus, gaining career-related resources to successfully manage, or craft, one’s early career is essential in today’s world of work (Akkermans and Tims, 2017). For young adults, crafting one’s career means adapting to the social environment while also finding ways to increase the chances of achieving one’s expectations when preparing for work roles (Savickas, 2005). The result of successfully doing so manifests itself in many forms, such as job, career or school satisfaction, performance, engagement, and life satisfaction (Rudolph et al., 2017). In the current study, we focus on two particular outcomes: students’ life satisfaction and academic performance. We chose life satisfaction as an outcome as it is at the core of optimal functioning and happiness of young adults (Proctor et al., 2009). Furthermore, we chose academic performance because it has been a central outcome in educational sciences, and has been shown to be a crucial predictor of long-term success (Richardson et al., 2012). Moreover, academic performance and life satisfaction complement each other as the former primarily assesses success in one’s study career, whereas the latter represents success in one’s broader life domain.

Quite a lot is known about the antecedents of life satisfaction and academic performance (e.g., Proctor et al., 2009; Richardson et al., 2012). However, up until now, it is still unclear which career resources – over and above study-related knowledge and skills – students need in order to safeguard their well-being and academic performance. Hirschi (2012) suggests that there are several types of resources that are, at least, necessary for successful career self-management, such as psychological (e.g., optimism, flexibility), identity (e.g., career adaptability), and social resources (e.g., networking). In previous scholarly work, there have been attempts to empirically investigate resources that contribute to young adults’ career success, putting forward important elements such as career planning and networking (De Vos et al., 2009), self-efficacy, optimism, and self-esteem (Bakker et al., 2015), and proactive career behaviors and hope (Hirschi, 2014). However, most of the research on career resources is performed using samples of employees, even though the preparation for the labor market is likely to start long before actually entering it.

In this contribution, we intend to fill this gap by examining two career resources that have recently received quite some attention in the career literature: career adaptability (Savickas, 2005) and career competencies (Akkermans et al., 2013a). Even though career adaptability and career competencies are related, both constructs conceptually and empirically differ from each other: career adaptability is primarily *reactive* in that it is about being able to adapt to constant changes, while career competencies are primarily *proactive* in that they are about achieving “person-career fit,” that is, developing competencies that help individuals to thrive in today’s dynamic labor market (Akkermans et al., 2015b). Thus, while mastering career adaptability resources allows individuals to effectively deal with situations that they face, career competencies prepare them for such situations by allowing to proactively craft their career (cf. Akkermans and Tims, 2017). Interestingly, although there is a conceptual difference between both types of career resources, they have not been empirically explored thus far. Hence, as an additional outcome of our study, we aim to provide preliminary empirical evidence of their unique added value as separate career resources. In all, in the present study, career adaptability and career competencies are investigated as two conceptually distinct but equally important career resources for students’ life satisfaction and academic performance.

Our study has three main contributions. First, this study adds to the literature by examining whether career adaptability and career competencies might be important career-related personal resources (hereafter referred to as career resources) for study success in today’s dynamic career landscape. While much prior research has focused on the attributes and skills that students must possess in order to enhance their chances for successful employment (Jackson and Chapman, 2012), in today’s highly demanding and competitive study and work context, not only the more distal outcome of employment success *after* graduating, but also more proximal outcomes like performance and satisfaction *during* their studies, are important. After all, these enable students to develop a sound foundation for starting off their career with strong human and psychological capital. Understanding if and how career resources enhance these outcomes is therefore crucial not only from an academic but also from a practical viewpoint. Second, we add to theorizing on the Job Demands-Resources (JD-R) theory (Demerouti et al., 2001; Bakker and Demerouti, 2017), which has thus far predominantly been applied to work settings, by examining whether career resources might be important antecedents in motivational processes among students. Third, this comprises, to the best of our knowledge, the first study incorporating career adaptability and career competencies in tandem, thereby enabling us to shed light on the similarities and differences between these two constructs.

### Job Demands-Resources Theory

The JD-R theory posits that every occupation consists of certain job characteristics that can be differentiated into two categories: job demands and job resources. Job demands, albeit not necessarily damaging, may result into strain when the employee requires high effort to meet these demands and cannot adequately recover from it (Boyd et al., 2011). Conversely, job resources are important means to either cope with job demands or comprise means that may help the employee to achieve or protect other valued resources. Otherwise stated, job resources may enhance well-being and motivation (Bakker and Demerouti, 2007). As such, JD-R theory is built upon two underlying psychological mechanisms that play a role in the development of job strain and motivation (Bakker and Demerouti, 2017): (1) a so-called health impairment process, and (2) a so-called motivational process. In both processes, personal resources – which can be characterized as a positive self-evaluation linked to resilience and a sense of control over one’s environment (Xanthopoulou et al., 2007) – are fundamental as they can buffer the potentially harmful effects of health impairment processes, and enhance the positive effects of motivational processes. In addition, similar to job resources, personal resources can initiate a motivational process that leads to enhanced individual outcomes – such as well-being and performance – via work engagement (Bakker and Demerouti, 2017) or job satisfaction (e.g., Akkermans et al., 2009). The importance of personal resources in promoting well-being and performance has been previously supported by a number of studies (e.g., Xanthopoulou et al., 2009; Salanova et al., 2010).

Though JD-R theory has mostly been explored in organizational contexts, some scholars have applied it to school (e.g., Salmela-Aro and Upadyaya, 2014) and university settings (e.g., Reis et al., 2015), and, previously, researchers have even referred to it as the Study Demands-Resources model (Mokgele and Rothmann, 2014). In this contribution, we aim to explain the potential value of career resources in promoting students’ well-being and academic performance through motivational processes. We apply the JD-R theory in an academic setting and expect that career adaptability and career competencies may function as career resources – a form of personal resources – that have predictive value for two important outcomes – students’ life satisfaction and academic performance – which are outcomes that have been related to JD-R processes in prior research (Bakker and Demerouti, 2014; Salmela-Aro and Upadyaya, 2014). More specifically, in the context of an academic setting, we propose that a motivational process occurs as a consequence of students’ career resources, which, in turn, results in positive outcomes.

### Career Adaptability and Career Competencies as Career Resources

As mentioned earlier, JD-R theory (Bakker and Demerouti, 2017) argues that personal resources are positive self-evaluations that are linked to resilience and a sense of control over one’s environment, which stimulate engagement, and help to manage one’s abilities. Individuals with higher levels of personal resources are motivated to pursue their goals, which triggers outcomes such as life satisfaction and performance (Bakker and Demerouti, 2014). For students, personal resources might serve as means of enhancing active learning behaviors and performance (Bakker et al., 2015), and for increasing mental health (Reis et al., 2015). Recent studies have shown that career adaptability (Maggiori et al., 2013) and career competencies (Akkermans et al., 2013b) can be considered as highly relevant personal resources. More specifically, these career resources can play an important role in motivational processes among young adults. Having higher levels of career competencies means having a clear idea about one’s preferences and wishes, and being able to set goals and to act upon these (Akkermans and Tims, 2017), while developing career adaptability enables individuals to manage present and impeding career challenges (Savickas, 1997) and to adjust one’s behaviors to the changing environment (Savickas and Porfeli, 2012). Thus, we propose that both career adaptability and career competencies may serve as career resources that are linked to desired outcomes via a motivational process in an academic setting. This is also in line with Career Construction theory (Savickas, 2005), which explains vocational development in nowadays’ world of work, and which states that individuals need to develop resources that anticipate changes in their future and its context (Savickas et al., 2009).

Career adaptability can be characterized as self-regulatory, psychosocial resources that condition the adapting strategies and behaviors while achieving adaptation goals (Savickas and Porfeli, 2012). It is essential for successful career self-management (Hirschi, 2012) and represents an individual’s readiness and resources for coping with vocational development tasks, occupational transitions and personal traumas (Savickas, 2005). Savickas and Porfeli (2012) differentiate between four career adaptability resources: *concern*, which is about becoming concerned about the career-related future, *control*, which focuses on taking control of trying to prepare for one’s vocational future, *curiosity*, which pertains to exploring possible selves and future scenarios, and *confidence*, which relates to strengthening the confidence to pursue one’s goals and aspirations).

Similar to career adaptability, career competencies are required for successful career management (Kuijpers et al., 2006; Hirschi, 2012), and can be defined as “knowledge, skills, and abilities that can be influenced and developed by the individual and are essential to career development” (Akkermans et al., 2013a, p. 246). Akkermans et al. (2013a) distinguish three dimensions of career competencies: reflective career competencies which include *reflection on motivation*, thus reflecting on values, passions, and motivations about one’s career, and *reflection on qualities*, meaning reflecting on one’s strengths, shortcomings, and skills); communicative career competencies include *networking*, which is the awareness of the presence and professional value of an individual network, and the ability to expand this network for career-related purposes, and *self-profiling*, thus presenting and communicating personal knowledge, abilities and skills to the labor market; and behavioral career competencies which include *work exploration*, in other words actively exploring and searching for career-related opportunities, and *career control*, which is actively influencing learning and work processes related to one’s personal career by setting goals and planning how to achieve them.

### The Role of Career Resources in Students’ Life Satisfaction

Life satisfaction is considered to be an important indicator of general well-being (Diener and Chan, 2011) and represents how individuals assess the overall satisfaction in their life according to own chosen criteria (Diener et al., 1985). An abundance of previous scholarly work has demonstrated that life satisfaction can predict health and longevity (Diener and Chan, 2011). Moreover, higher life satisfaction among students can be achieved by procrastinating less, by having higher self-control, and by applying more mastery-approach goals (Howell, 2009). Consequently, because of its central role in prior research, and due to the major changes in students’ early careers, as described earlier, life satisfaction is an important way of gauging students’ general well-being and enhances their chances for career success once entering the labor market and beyond (see also Boehm and Lyubomirsky, 2008).

We posit that life satisfaction can be considered to be an outcome of motivational processes (Bakker and Demerouti, 2014). In particular, in line with the JD-R theory (Bakker and Demerouti, 2017), personal resources are related to outcomes, such as well-being, via a resource building process. Having more resources means that individuals can become more enthusiastic and active in changing their environment, which, in turn, leads to more satisfying outcomes of their actions and a better mental health. In addition, people with more resources are more likely to be able to cope with stressful circumstances that may negatively affect their psychological and physical well-being (Hobfoll, 2002). Hence, it is likely that personal resources – in our case, career resources – are related to life satisfaction.

Indeed, previous research has argued that career adaptability and career competencies are related to life satisfaction. For example, King (2004) suggested and found that various career self-management behaviors related to life satisfaction. Similarly, career adaptability has been shown to be linked to life satisfaction of both students (Buyukgoze-Kavas et al., 2015) and adults (Maggiori et al., 2013). In addition, Akkermans and Tims (2017) reported that career competencies were indirectly related to career success via a motivational process. Based on their findings, it would be likely that young adults who learn how to reflect on their passions and qualities are likely to be more satisfied with their overall well-being, and those who actively seek for and find developmental opportunities might also be generally more satisfied with their life. Therefore, in line with JD-R theory and findings from earlier empirical work, we propose the following:

Hypothesis 1: Career resources in the form of (a) career adaptability and (b) career competencies will be positively related to students’ life satisfaction.

### The Role of Career Resources in Students’ Academic Performance

An important indicator of students’ academic success is their academic performance, which is most often expressed in terms of Grade Point Average (GPA), that is, the weighted mean score of the grades from all courses contributing to the assessment of the final degree (Richardson et al., 2012). Research has shown that students with poor academic performance can exhibit low participation, high levels of procrastination – although this particular relationship varies based on the type of measurement used (Kim and Seo, 2015) – and study withdrawal, poor results, and academic failure (Infante and Marín, 2008). At the same time, good academic performance, in terms of grades, is still widely used as a selection criterion when employers are hiring graduating students applying for their first job (Robbins et al., 2004). It has also been linked to a higher starting salary at work (Vermeulen and Schmidt, 2008) and enhanced socio-economic success (Strenze, 2007). In today’s more dynamic educational world and labor market, career-relevant experiences and skills may be crucial in the long-term development and performance of students, starting during their studies (Lent and Brown, 2013). We argue that career adaptability and career competencies might be two important career resources linked to students’ academic success.

Resources are valuable means, and they motivate people to invest those resources (e.g., energy) to gain additional resources (e.g., reputation, money), subsequently contributing to better functioning (Hobfoll, 2002). JD-R theory also states that personal resources can lead to enhanced organizational outcomes in the form of performance via motivational processes (Bakker and Demerouti, 2007). In line with this reasoning, it is likely that higher levels of career resources would directly contribute to performance (e.g., Judge et al., 2004). For example, if students learn how to communicate and network effectively, and if they are able to take control over their vocational future, it would make sense that their enhanced social capital and deliberate actions toward their future would result in increased academic performance. Similarly, if students actively set goals and strive to fulfill them, their performance would likely increase as a result. In fact, it has been previously demonstrated that career adaptability contributes to improved academic performance (i.e., GPAs) in samples of undergraduate students (Rottinghaus et al., 2005) and adolescents (Negru-Subtirica and Pop, 2016), and that career competencies can enhance employee career success (Akkermans and Tims, 2017). To sum up, based on the propositions of the JD-R theory and on previous research linking career resources to performance outcomes, we propose the following:

Hypothesis 2: Career resources in the form of (a) career adaptability and (b) career competencies will be positively related to academic performance.

As outlined above, the JD-R theory states that personal resources are indirectly related to distal outcomes via motivational processes. Therefore, we present two specific mediators in the relationships between career resources and student outcomes: academic major satisfaction and study engagement.

### The Mediating Role of Academic Major Satisfaction and Study Engagement

In line with JD-R theory, career resources are reasoned to be at the start of a resource building process, in which an increase in available resources enables individuals to become more satisfied and engaged (cf. Akkermans and Tims, 2017). Consequently, these higher levels of satisfaction and engagement can contribute to improved individual and organizational outcomes. In parallel with job satisfaction, being considered in JD-R theory as a mediator in the motivational process in work settings (Akkermans et al., 2009), it would make sense that academic major satisfaction, which represents global satisfaction with one’s chosen major and unwillingness to change it (Nauta, 2007), might work in a similar way for students. Similarly, work engagement has been extensively used as a mediator in JD-R theory (Bakker and Demerouti, 2017) and, therefore, we argue that study engagement might be part of a motivational process between resources and students’ outcomes as well (Salanova et al., 2010; Salmela-Aro and Upadyaya, 2014). Hence, based on these premises, we assume that career adaptability and career competencies – being career resources – are likely to enhance students’ academic major satisfaction and study engagement, which would then be associated with enhanced life satisfaction and academic performance.

Previous studies at least partially support this notion. In terms of academic major satisfaction, this variable has been shown to be impacted by career adaptability (Duffy et al., 2015; Urbanaviciute et al., 2016) and a variety of career management behaviors (Cox et al., 2015), and it appears to be related to life satisfaction (Sovet et al., 2014) and academic performance (McIlveen et al., 2013). Similarly, career adaptability (e.g., Rudolph et al., 2017) and career competencies (e.g., Akkermans et al., 2013b) appear to enhance work engagement, and, in the context of our academic setting, study engagement has been shown to relate to students’ life satisfaction (Mokgele and Rothmann, 2014) and to academic performance (Salanova et al., 2010). To illustrate, students who actively set goals, take control over their vocational future, and those who are more confident to pursue their aspirations, are likely to become more satisfied with their study program, more dedicated to their studies and to feel more absorbed by them. In turn, this enhanced engagement in their studies would generally allow them to experience a better well-being and to achieve higher performance. Hence, based on the JD-R theory and previous empirical findings, we hypothesize the following:

Hypothesis 3: Academic major satisfaction will mediate the positive relationship between career resources and (a) life satisfaction and (b) academic performance.
Hypothesis 4: Study engagement will mediate the positive relationship between career resources and (a) life satisfaction and (b) academic performance.

## Materials and Methods

### Participants and Procedure

Paper-and-pencil survey questionnaires were administered by the primary researchers responsible for this project, which were completed during regular lecture hours. With regard to research ethics, we did not seek approval from an ethical committee as the survey research that we performed was exempt from such approval in the country in which the study was performed (i.e., Lithuania) and by the institutions leading this project. All research participants were informed in an introductory explanation of the survey that they would formally agree to participate in the research by filling out the survey, thereby giving informed consent if they chose to participate. All participants were informed that their participation was completely voluntary and that they could quit at any time.

Data for this study were collected in Lithuania. According to data collected by the Lithuanian Statistics Department in 2015, up to 70% of high school graduates enter universities or colleges right after finishing high school. In Lithuania, students have to choose their specialty before they apply for higher education institutions. Usually, young adults can receive career counseling in high school before choosing their specialization. In addition, there are some national career guidance programs in schools. Finally, some universities and colleges have career guidance centers, which provide information on how to find a job, and offer courses on how to prepare for the labor market, yet, attending those course or career guidance activities is optional.

In total, 950 Bachelor students from nine Lithuanian educational institutions participated in the study. The participating institutions were located in Vilnius, Kaunas and Klaipeda, three of the biggest cities of the country. Of the 950 surveys, 25 came back blank, and another 65 surveys were incomplete. Finally, only those students who answered the question about their average grade were included in the study, resulting in a final sample size of 672 participants. Our final sample consisted of 213 males (31.70%), and the age ranged from 18 to 29 years (*M* = 20.62, *SD* = 1.70). The majority of the participants (*n* = 532; 79.20%) consisted of university students, and the remaining 20.80% (*n* = 140) were students from college. The final sample consisted of 235 (35.0%) 1st-year students, 200 (29.8%) 2nd-year students, and 237 (35.3%) last year students. In terms of study programs, 78 (11.6%) participants were students of technological sciences, 426 (63.4%) participants were students of social sciences, 107 (15.9%) were students of biomedical sciences, 20 (3.0%) were students of physical sciences, 32 (4.8%) were students of humanitarian sciences, and 9 (1.3%) were students of arts.

### Measurement Instruments

Below, an overview of the measurement instruments is given. Please note that we assessed career resources as *perceptions* of those resources, as is the standard in scholarly research on career adaptability (Savickas and Porfeli, 2012; Maggiori et al., 2013) and career competencies (Akkermans et al., 2013a). Also, the Cronbach alpha values that we report are from the current study, thus representing the reliabilities of the Lithuanian versions of the scales. These items were created using the translation back-translation method (Brislin, 1970; Beaton et al., 2000). The original scales have all been extensively validated in prior research (references are provided below for each instrument).

#### Career Adaptability

Career adaptability was measured with the 12-item *Career Adapt-Abilities Scale – Short Form* (CAAS-SF; Maggiori et al., 2017), consisting of *concern* (3 items, e.g., “Thinking about what my future will be like,” α = 0.79); *control* (3 items, e.g., “Taking responsibility for my actions,” α = 0.78); *curiosity* (3 items, e.g., “Investigating options before making a choice,” α = 0.74); and *confidence* (3 items, e.g., “Taking care to do things well,” α = 0.76). All items were scored on a five-point Likert scale ranging from 1 (*not a strength*) to 5 (*greatest strength*). The outcomes of elaborate psychometric tests support the validity of using the short-form as a pertinent and economic alternative of the 24-item original version (see Maggiori et al., 2013).

#### Career Competencies

Career competencies was measured with the 21-item *Career Competencies Questionnaire (CCQ*; Akkermans et al., 2013a) consisting of *reflection on motivation* (3 items, e.g., “I know what is important to me in my career,” α = 0.82); *reflection on qualities* (4 items, e.g., “I know which skills I possess,” α = 0.80); *networking* (4 items, e.g., “I know how to ask for advice from people in my network,” α = 0.79); *self-profiling* (3 items, e.g., “I can clearly show others what my strengths would be in my work,” α = 0.81); *work exploration* (3 items, e.g., “I am able to explore my possibilities on the labor market,” α = 0.74); and *career control* (4 items, e.g., “I am able to set goals for myself that I want to achieve in my career,” α = 0.85). All items were scored on a five-point Likert-type scale ranging from 1 (*completely disagree*) to 5 (*completely agree*). The CCQ has received elaborate psychometric support (e.g., Akkermans et al., 2013a,b, 2015a).

#### Life Satisfaction

Life satisfaction was measured with the *Satisfaction With Life Scale* (SWLS; Diener et al., 1985) that consists of five items (e.g., “I am satisfied with my life,” α = 0.86). Participants responded to items using a 7-point Likert scale ranging from 1 (*strongly disagree*) to 7 (*strongly agree*). The scale shows good convergent validity with other scales and with other types of assessments of subjective well-being (Pavot and Diener, 1993).

#### Academic Performance

Academic performance was measured by asking students to report their GPA of the previous semester. In the Lithuanian grading system, grades range from 1 (*low*) to 10 (*high*), with grades lower than 5 implying a failed exam.

#### Academic Major Satisfaction

Academic major satisfaction was measured with the *Academic Major Satisfaction Scale* (AMSS; Nauta, 2007). The scale consists of six items (e.g., “Overall, I am happy with the major I’ve chosen,” α = 0.90), with a 5-point Likert-type scale ranging from 1 (*strongly disagree*) to 5 (*strongly agree*). Exploratory and confirmatory factor analyses (CFA) of the original scale suggested that it has a reliable unidimensional structure. Moreover, it appeared to distinguish between students who remained in their major vs. those who changed major during 1- and 2-year periods (Nauta, 2007).

#### Study Engagement

Study engagement was measured with the 9-item short-form version of the *Utrecht Work Engagement Scale – student version* (UWES-S-9; Schaufeli et al., 2006) consisting of (a) *vigor* (3 items, e.g., “When I’m doing my work as a student, I feel bursting with energy,” α = 0.80), *dedication* (3 items, e.g., “My study inspires me,” α = 0.86), and *absorption* (3 items, e.g., “I am immersed in my studies,” α = 0.72). All items were scored on a seven-point Likert scale ranging from 0 (*never*) to 6 (*always/every day*). The psychometric qualities of the measure are adequate (Schaufeli et al., 2006; Salmela-Aro and Kunttu, 2010).

To assure that the results were not affected by demographic differences of the rather heterogeneous sample, we included several control variables. Similar previous studies have used age and gender (e.g., Urbanaviciute et al., 2016) as control variables. Hence, we included these as controls in our study too. Also, we included study year and educational institution type as control variables.

### Strategy of Analysis

Before testing the hypothesized model, we examined the measurement models in which variables were modeled as latent factors with their items or scale means as indicators of the latent constructs. Confirmatory factor analyses using maximum likelihood estimation in Mplus 7 (Muthén and Muthén, 2012) were performed to check the measurement models. Model fit was ascertained using various indices: the Comparative Fit Index (CFI) and the Tucker-Lewis Index (TLI) should exceed 0.90, the Root Mean Square Error of Approximation (RMSEA) should be less than 0.08, and Standardized Root Mean Square Residual (SRMR) should be less than 0.05 (Byrne, 2012). Specifically, the latent factor career competencies was modeled with the six scale means as indicators; career adaptability was modeled with the four scale means as indicators; life satisfaction was modeled with the five items as indicators; academic performance was modeled as an observed variable; academic major satisfaction was modeled with the six scale means as indicators; and study engagement was modeled with the three scale means as indicators. Latent scores for the total variables – with their dimensions or parcels as indicators – were used for three reasons: first, because this is commonly done both from a conceptual and empirical perspective in prior research on these variables (e.g., Hirschi, 2009; Akkermans and Tims, 2017), second, because we had no theoretical reasons to expect different relationships depending on the sub-dimension of these constructs, and third, to make the model as parsimonious as possible. Next, we examined the hypothesized model using latent variable path analyses with Structural Equation Modeling (SEM). The mediating role of study engagement and academic major satisfaction was tested using the bootstrap option (2,000 bootstrap samples).

## Results

### Descriptive Statistics

The descriptive statistics and correlations can be found in **Table 1**. In general, the correlations were in the expected direction. Of the demographics, age was only related with GPA (*r* = 0.15, *p* < 0.001) and career competencies (*r* = 0.09, *p* = 0.023). In addition, we checked the differences between students of different gender, study year, and education institution type, and these all appeared to play a minimal role in explaining the study variables. Full details about these differences can be obtained from the corresponding author.

**Table 1:** Correlations between study variables, means, and standard deviations.

		*M*	*SD*	1	2	3	4	5	6	7
1	Age	20.62	1.70	1.00						
2	Career adaptability	44.27	7.69	0.02	1.00					
3	Career competencies	3.70	0.55	0.09*	0.57**	1.00				
4	Life satisfaction	23.90	6.31	-0.02	0.42**	0.39**	1.00			
5	GPA	8.00	1.02	0.15**	0.11**	0.12**	0.10*	1.00		
6	Academic major satisfaction	3.90	0.87	-0.02	0.29**	0.21**	0.42**	0.18**	1.00	
7	Study engagement	3.35	1.08	-0.03	0.39**	0.33**	0.49**	0.25**	0.65**	1.00


**N = 672; ^∗^p < 0.05; ^∗∗^p < 0.01. Mean scores of career adaptability and life satisfaction represent sum scores as dictated by the original survey descriptions.**

### Testing the Measurement Models

First, we tested the measurement models with all study variables included, but without specifying any structural paths. For career competencies, modeled with six indicators, after specifying a correlation between reflection on qualities and reflection on motivation (which is conceptually acceptable and has been done before, see e.g., Akkermans and Tims, 2017) there was a satisfactory fit to the data (χ^2^ = 44.49, *p* < 0.001, *df* = 8, χ^2^/*df* = 5.56, CFI = 0.97, TLI = 0.95, RMSEA = 0.08, SRMR = 0.03). Career adaptability (χ^2^ = 88, *p* = 0.643, *df* = 2, χ^2^/*df* = 0.44, CFI = 1.00, TLI = 1.00, RMSEA = 0.00, SRMR = 0.01), life satisfaction (χ^2^ = 28.14, *p* < 0.001, *df* = 5; χ^2^/*df* = 5.63; CFI = 0.99, TLI = 0.97; RMSEA = 0.08; SRMR = 0.02) and academic major satisfaction also fitted adequately to our data (χ^2^ = 22.93, *p* = 0.002, *df* = 7; χ^2^/*df* = 3.28; CFI = 0.99, TLI = 0.99; RMSEA = 0.06; SRMR = 0.02). Finally, study engagement was measured with three indicators and had a perfect model fit, irrespective of the pattern of factor loadings, which happens to just-identified models (Byrne, 2012). Factor loadings for all the study variables ranged between 0.57 and 0.95. We ran one additional CFA on the items belonging to both “career control” variables in the CAAS and CCQ. Although they have the same name, both dimensions are conceptually different, but we wanted to make sure that they are also empirically different. To test this, we conducted a CFA in which two models were compared: one in which all items of these two factors loaded onto a single latent factor, and another one in which they loaded onto two separate factors. The first model showed a poor fit to the data (χ^2^ = 524.68, *df* = 14, CFI = 0.72, TLI = 0.58, RMSEA = 0.23). However, the model in which the items loaded onto their intended factors showed a significantly better fit (Δχ^2^ = 489.61, Δ*df* = 1, *p* < 0.001, CFI = 0.99, TLI = 0.98, RMSEA = 0.05). These results provide evidence for the two career control resources being different both conceptually and empirically.

Next, based on the distinguished separate measurement models, we tested the overall measurement model, which included all study variables at once. The final measurement model included career competencies (six subscales as indicators), career adaptability (four subscales as indicators), academic major satisfaction (six scale items as indicators), study engagement (three subscales as indicators), life satisfaction (five scale items as indicators), and GPA as six separate factors. This model demonstrated an acceptable fit to the data (χ^2^ = 793.54, *p* < 0.01, *df* = 261, χ^2^/*df* = 3.04, CFI = 0.93, TLI = 0.93, RMSEA = 0.05, SRMR = 0.04).

To further examine whether the proposed measurement model had the best possible fit and to make sure that common-method variance was not an issue (Podsakoff et al., 2003), we tested three alternative models. First of all, we collapsed all factors into one latent factor. This model showed a significantly worse fit compared with the final measurement model (Δχ^2^ = 3444.25, Δ*df* = 14, *p* < 0.001). Next, we tested a five-factor model wherein four career adaptability indicators and six career competencies indicators were collapsed into one latent factor, whilst academic major satisfaction, study engagement, life satisfaction, and GPA were incorporated as separate factors. This model also fitted the data significantly worse than the six-factor measurement model (Δχ^2^ = 307.14, Δ*df* = 5, *p* < 0.001). Finally, to make sure that academic major satisfaction and study engagement were two separate constructs, we tested a model wherein these two constructs were collapsed, leaving career adaptability, career competencies, life satisfaction, and GPA as separate factors. This model also fitted the data significantly worse in comparison with the six-factor model (Δχ^2^ = 543.13, Δ*df* = 5, *p* < 0.01). Hence, our hypothesized measurement model showed the best fit to the data.

### Testing the Hypotheses

**FIGURE 1:**
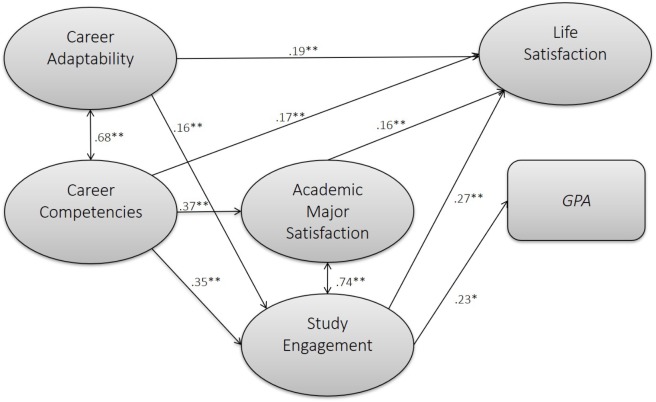
Results of empirical model test (only significant paths depicted).

Fit indices of the overall hypothesized structural model showed that the model fitted the data adequately (χ^2^ = 793.54, *p* < 0.001, *df* = 261, χ^2^/*df* = 3.04, CFI = 0.94, TLI = 0.93, RMSEA = 0.06, SRMR = 0.04; **Figure 1** shows the results).

In line with our hypotheses, both career adaptability (β = 0.19, *p* < 0.01) and career competencies (β = 0.17, *p* < 0.01) were positively related to life satisfaction, thereby supporting Hypotheses 1a and 1b. However, contrary to what was expected, neither career adaptability (β = 0.06, *p* = 0.34) nor career competencies (β = -0.02, *p* = 0.82) were significantly related to students’ GPAs. Hence, we could not find support for Hypotheses 2a and 2b.

Next, we tested potential mediation effects of academic major satisfaction and study engagement. First, career adaptability was not significantly related to academic major satisfaction, and the indirect effects between career adaptability and outcome variables via academic major satisfaction were non-significant. Career competencies were positively related to academic major satisfaction (β = 0.37, *p* < 0.001) and academic major satisfaction was subsequently linked to life satisfaction (β = 0.16, *p* = 0.009). In addition, academic major satisfaction was a significant mediator between the two constructs (standardized indirect effect = 0.06, *p* = 0.017). However, academic major satisfaction was neither related to students’ GPAs nor acted as a significant mediator between career competencies and academic performance (see **Table 2**). Thus, we found partial support for Hypothesis 3a for career competencies but not for career adaptability, and no support for Hypothesis 3b.

**Table 2:** Indirect effects of career adaptability and career competencies on outcome variables.

Variables	Estimate	SE	95% BC CI	
			
			Lower	Upper
**Career adaptability → life satisfaction**		
Via study engagement	0.04*	0.02	0.01	0.08
Via academic major satisfaction	-0.002	0.01	-0.02	0.02
Total indirect effect	0.04	0.03	-0.01	0.09
**Career competencies → life satisfaction**		
Via study engagement	0.09**	0.03	0.04	0.14
Via academic major satisfaction	0.06*	0.03	0.01	0.11
Total indirect effect	0.15**	0.03	0.10	0.21
**Career adaptability → GPA**		
Via study engagement	0.04*	0.02	0.01	0.07
Via academic major satisfaction	0.00	0.01	-0.01	0.01
Total indirect effect	0.04	0.02	0.00	0.07
**Career competencies → GPA**		
Via study engagement	0.08**	0.03	0.03	0.13
Via academic major satisfaction	0.001	0.03	-0.04	0.05
Total indirect effect	0.08**	0.02	0.04	0.12


**SE, standard error; BC CI, bias-corrected confidence intervals. ^∗^p < 0.05, ^∗∗^p < 0.01.**

Finally, study engagement mediated the relationship between career adaptability and life satisfaction (standardized indirect effect = 0.04, *p* < 0.05), and between career adaptability and GPA (standardized indirect effect = 0.04, *p* < 0.05). In addition, study engagement was a significant mediator between career competencies and life satisfaction (standardized indirect effect = 0.09, *p* < 0.01), and between career competencies and GPA (standardized indirect effect = 0.08, *p* < 0.01). These findings provide full support for Hypotheses 4a and 4b. It is important to note that study engagement was more strongly related to career competencies (β = 0.35, *p* < 0.01) than to career adaptability (β = 0.16, *p* < 0.01). In addition, the indirect effects between career competencies and the outcome variables were stronger than between career adaptability and the outcome variables.

Finally, we tested whether the models would differ when we included the control variables in the model. In terms of measurement invariance, following the suggestions of Vandenberg and Lance (2000) we tested for both configural and measurement invariance. We included age, gender, study year, and educational institution type as control variables in this analysis. In order to determine possibly significant differences between the models, we followed Chen (2007) recommendations, in which it is suggested that ΔCFI ≥ 0.01 and ΔRMSEA ≥ 0.015 would indicate non-invariance. The findings indicate that the models in which the regression coefficients were free between groups were not significantly different from the models where the regression coefficients were fixed to be equal when controlling for age (ΔCFI = 0.000, ΔRMSEA = 0.000), gender (ΔCFI = 0.002, ΔRMSEA = 0.000), study years (ΔCFI = 0.000, ΔRMSEA = 0.000), and educational institution type (ΔCFI = 0.000, ΔRMSEA = 0.000). Gender was the only variable where ΔCFI and ΔRMSEA were not consistent. Chen (2007) suggested that a number of factors can affect the magnitude of changes in fit statistics, such as pattern of non-invariance, sample size, ratio of sample size, and model complexity. It is possible that, in our empirical study, ΔCFI was larger in the case of gender, because the accompanying sample sizes were unequally distributed: only 213 participants (31.7%) were male, meaning that the sample of females was twice as large. Full details of the invariance analysis can be found in **Table 3**. These results indicate that the final model fits the data comparably across the control variables, herewith supporting that the results of the path analyses are similar between students with different demographical backgrounds.

**Table 3:** Results of invariance analyses of the final hypothesized model.

Model	χ^2^	*df*	χ^2^/*df*	CFI	TLI	RMSEA [90% CI]	SRMR	ΔCFI	ΔRMSEA
**Testing gender invariance**		
Configural invariance	481.36^∗∗^	204	2.36	0.94	0.93	0.06 [0.06; 0.07]	0.05	–	–
Metric invariance^a^	507.66^∗∗^	218	2.32	0.94	0.93	0.06 [0.06; 0.07]	0.06	0.002	0.000
**Testing invariance according to study year**		
Configural invariance	659.24^∗∗^	344	1.91	0.93	0.93	0.064 [0.06; 0.07]	0.08	–	–
Metric invariance^a^	623.18^∗∗^	316	1.97	0.93	0.93	0.066 [0.06; 0.07]	0.06	0.000	0.000
**Testing invariance according to students’ age^b^**		
Configural invariance	1480.89^∗∗^	859	1.72	0.93	0.93	0.057 [0.052; 0.062]	0.07	–	–
Metric invariance^a^	1540.99^∗∗^	887	1.74	0.93	0.93	0.057 [0.053; 0.062]	0.09	0.000	0.000
**Testing invariance according to education institution type**		
Configural invariance	470.44^∗∗^	204	2.31	.94	0.93	0.06 [0.06; 0.07]	0.06	–	–
Metric invariance^a^	483.39^∗∗^	218	2.22	0.94	0.94	0.06 [0.05; 0.07]	0.07	0.000	0.000


*χ^2^*, *chi-square; df, degrees of freedom; CFI, comparative fit index; TLI, Tucker–Lewis index; RMSEA, root mean square error of approximation; SRMR, standardized root mean square residual. ^*a*^Compared to configural invariance. ^*b*^Students were divided into three age groups: 1 (<M - 1SD), 2 (M ± 1SD), 3 (>M + 1SD). ^∗∗^p < 0.01.*

## Discussion

In order to achieve well-being and long-term career success in today’s educational world and dynamic labor market, which are characterized by an increasing emphasis on individual agency (De Vos et al., 2018), it has become crucial that young individuals not only focus on gaining study-related knowledge and skills but that they already start developing career-related knowledge and skills as well. Hence, students need to build up career-related resources to make sure that their well-being and academic performance are high, ultimately enabling them a successful transition into the labor market. Using the Job Demands-Resources (JD-R) theory (Demerouti et al., 2001; Bakker and Demerouti, 2017) as our conceptual framework, we examined whether career resources (i.e., career adaptability and career competencies) would be related to students’ life satisfaction and academic performance via academic major satisfaction and study engagement.

### Main Results and Theoretical Implications

In line with our expectations, career adaptability and career competencies were positively related to life satisfaction, both directly and indirectly via study engagement. In addition, career adaptability and career competencies were indirectly related to academic performance via study engagement. Finally, career competencies were indirectly related to life satisfaction via academic major satisfaction. In line with earlier research (Akkermans et al., 2013b), these results show that career adaptability and career competencies are both important career resources that can enhance student well-being and performance. According to JD-R theory (Bakker and Demerouti, 2014, 2017), personal resources are indirectly related to distal outcomes via motivational processes. Our results confirm this theoretical notion using career adaptability and career competencies as career-related personal resources. In addition, our results expand existing findings about motivational processes (Salanova et al., 2010), by showing that such processes also occur in academic settings, that is among students, using study engagement – and, to a lesser degree, academic major satisfaction – as a central mediator, and with career resources as their foundation (cf. Salmela-Aro and Upadyaya, 2014; Reis et al., 2015).

It is interesting to note that we found mixed results with regard to the mediating role of academic major satisfaction. In line with our expectations, academic major satisfaction mediated the positive relationship between career competencies and life satisfaction. However, we did not find support for its mediating role in the relationship between career adaptability and life satisfaction. In addition, academic major satisfaction did neither mediate the relationship between career adaptability and academic performance nor between career competencies and academic performance. Thus, even though there was some support for satisfaction with one’s major as a central activating mechanism in motivational processes, when studying the role of career competencies in young adults’ well-being (cf. Akkermans et al., 2009), this evidence was mixed at best, and did not hold when examining career adaptability as a career resource. Our study indicates that while academic major satisfaction plays a role in the light of enhancing students’ life satisfaction, their study engagement appears to be key in case their academic performance need to be improved.

To elaborate on this in more detail, considering our findings that study engagement did consistently act as a mediator, and considering previous theorizing and empirical evidence that work/study engagement is at the core of motivational processes (Salanova et al., 2010; Christian et al., 2011; Bakker and Demerouti, 2014), we argue that this also applies to a student context. Hence, from this contribution we may conclude that study engagement seems to be a stronger mobilizer of career resources among students, compared to the satisfaction with one’s choice for study program. This outcome adds to previous findings (e.g., Alarcon and Lyons, 2011) that being actively vigorous, dedicated, and absorbed is more important for well-being and performance than being satisfied with one’s job or study.

Furthermore, although we expected that career resources would be positively related to academic performance, both directly and indirectly, we only found indirect relationships. Interestingly, this implies that in spite of the fact that career resources can directly impact students’ well-being in the form of life satisfaction, these resources need to be mobilized through an active motivational state – here: study engagement – before they can result in improved academic performance. An explanation for this finding may be that academic performance is predicted by factors that are more proximally related to it (Salanova et al., 2010), such as study motivation and engagement, whereas well-being might also be directly influenced when students become more resilient, aware of themselves, and feel more in control (i.e., possess career resources). Hence, it is plausible that when students gain career resources, this initiates a motivational process in which they first become more vigorous, dedicated, and absorbed with their studies, and consequently perform better in terms of higher grades. Taken together, these findings support the applicability of the JD-R theory (Bakker and Demerouti, 2017) in study settings by showing that career resources, in the form of career adaptability and career competencies, might enhance study performance indirectly via a motivational process through study engagement.

To the best of our knowledge, this study is one of the first attempts to introduce both career adaptability and career competencies in one empirical study. Both constructs have been popular themes in the recent scholarly debate regarding young adults’ career management, yet their simultaneous effects have rarely been explored theoretically or empirically up until now (for an exception of a conceptual comparison, see Akkermans et al., 2015b). On the one hand, our study shows that career adaptability and career competencies may be quite similar constructs that both have an important role to play as career resources in young adults’ well-being and study performance. These career resources might initiate motivational processes among students and young adults in general. Yet, our study also confirms that career adaptability and career competencies are clearly different factors. From a conceptual perspective, this makes sense as career adaptability predominantly relates to gathering resources to deal with certain career events (Hirschi et al., 2015), whereas career competencies focus more on knowledge and abilities that help to proactively shape one’s career (Kuijpers et al., 2006; Akkermans et al., 2013a). We provide empirical evidence to support this notion. First, after conducting CFAs and testing our measurement models, we showed that career adaptability and career competencies are indeed clearly distinct constructs. Second, the outcomes of our empirical work show that only career competencies were significantly related to academic major satisfaction, and indirectly to life satisfaction via academic major satisfaction, whereas career adaptability was not. Third, our findings show that career competencies were more strongly related to study engagement than career adaptability. Finally, the total indirect effects through the incorporated mediators were higher in the case of career competencies compared to career adaptability. Hence, although both career adaptability and career competencies seem important career resources in enhancing student well-being and performance, our results suggest that career competencies may have a slightly stronger role to play.

### Limitations and Directions for Future Research

Our study has several key strengths, such as comprising a large Lithuanian sample from nine different higher education institutions, robust statistical analyses, and the application of JD-R theory – which has been predominantly tested in work settings – in an academic context using a sample of students. Yet, there were also several limitations that may be translated into ideas for future research. First, we used a cross-sectional design, and future work conducting longitudinal approaches might add to our understanding. After all, career resources might change over time and there is a possibility of reciprocity between resources, as the existence of resources promotes the development of additional resources (Hobfoll, 2002). A cross–lagged analysis with several measurements over time could allow investigation of reciprocal relationships between study variables. For example, students could be tracked over time during their studies, thereby examining whether career resources at the beginning of the study program might influence well-being and academic performance at the end. Alternatively, a fascinating possibility would be to study whether these career resources would prepare students better for the actual transition into the labor market by using a longitudinal design comparing students before and after this transition.

A second limitation of our study is that due to the use of self-report measures common method variance may have been an issue. However, following Podsakoff et al. (2003) we performed several statistical tests to check whether this was an issue and we did not find any serious limitations. Also, it is important to note that, although except for the GPA scores, our study variables are almost impossible to be measured otherwise than through self-perceptions (Mäkikangas et al., 2004), it would be important to also incorporate more objective measures in future research, for example by measuring career resources with multi-source data (e.g., peers, teachers or career counselors) or reporting actual competencies and behaviors (e.g., do they actually explore their possibilities on the labor market or do they actually ask for advice from people in their network) as well. Such data would complement the information obtained in thus study, which was based upon the established conceptualizations and measurement of career adaptability and career competencies as self-perceived career resources. In line with the current debate in the field of employability, there might be different constructs at play here – for example actual career adaptability vs. perceived career adaptability – that can influence each other over time (cf. Forrier et al., 2015). Future research might shed additional light on this possibility.

Third, our study focused exclusively on career resources and motivational processes, thereby excluding potential study demands and health impairment processes, which are a seminal part of JD-R theory as well (Bakker and Demerouti, 2014). Previous research has already shown that students can face highly demanding situations and sometimes even drop out of their studies because of, for example, a high workload (Whitelock et al., 2015). Indeed, earlier work that included study demand-related variables, such as workload and professional self-doubt, has shown that these are associated with study engagement and burnout (Robins et al., 2015). According to Bakker and Demerouti (2017), certain interplays between the components of JD-R theory have not been thoroughly explored and explained yet. Therefore, one interesting avenue for future research would be to study whether career resources might also serve as buffers against negative effects of high study demands.

Finally, future research could test whether career resources might benefit students in other ways than their life satisfaction and academic performance. In terms of study success, it would, for example, be interesting to examine whether career resources might enhance students’ proactive behaviors, such as job crafting, and thereby their study success. A recent study by Akkermans and Tims (2017) showed that young workers with increased career resources would subsequently craft their jobs, and, as a result, experience more career success. A parallel could be drawn with regard to the notion of ‘study crafting’, where students who develop career resources might proactively increase their study resources (e.g., social support, feedback) and reduce their study demands (e.g., work pressure, conflicts), herewith ultimately increasing their well-being and performance. In terms of long-term outcomes, it would be important for future research to examine school-to-work transitions (e.g., Ryan, 2001), starting during the study period and continuing during working life. In this way, future research could shed more light on whether career resource building during the study period might enable students to achieve more long-term career success after the transition into working life as well.

### Practical Implications

We would like to advise educational institutions to build programs wherein students are trained to further develop their career resources, over and above their study curriculum. Previous studies have already shown that these career resources are malleable and can be enhanced after targeted interventions. For example, a 1 day group training aimed at increasing graduates’ capabilities was shown to enhance career adaptability and to lead to better subsequent employment quality (Koen et al., 2012). Similarly, the CareerSKILLS program, a career development intervention program that is aimed to stimulate young adults’ career potential, has been shown to increase young employees’ career competencies, their work engagement and employability (Akkermans et al., 2015a). Related to this, career construction counseling might also be beneficial in helping students to manage their early career (Cardoso et al., 2014) as it promotes both career adaptability and subjective well-being (Hartung and Taber, 2008). In all, we would like to advise higher education institutions to implement various interventions and counseling strategies focusing on enhancing students’ career resources, as this might have added value both for their well-being and their studies.

A second practical implication of our study is that we emphasize the importance of enhancing student engagement. Engagement in learning activities is malleable as well and educators may be able to influence this important characteristic in order to increase students’ chances of completing their education successfully (Bakker et al., 2015). Indeed, a recent meta-analysis showed that, although the effects are usually modest, specific interventions can enhance student engagement (Knight et al., 2017). For example, a 4 months individual cognitive-behavioral intervention program building upon Social Cognitive theory appeared to increase students’ engagement, self-efficacy, and performance (Bresó et al., 2011). Though positive interventions might be beneficial to all possible participants, some strategies might be even more beneficial for those who lack work engagement, compared to those who are already engaged (Ouweneel et al., 2013). Hence, educators should keep in mind that this might also be true for higher education settings and that it is important to tailor interventions and workshops to different students with varying levels of study engagement.

## Data Availability

The raw data supporting the conclusions of this manuscript will be made available by the authors, without undue reservation, to any qualified researcher.

## Author Contributions

JA contributed in coordination, theorizing, analyzing, and writing. KP contributed in data collection, analyzing, theorizing, and writing. BVdH and ADV contributed in theorizing and writing.

## Conflict of Interest Statement

The authors declare that the research was conducted in the absence of any commercial or financial relationships that could be construed as a potential conflict of interest.

## References

[B1] Akkermans J. Brenninkmeijer V. Blonk R. W. B. Koppes L. L. J. (2009). Fresh and healthy? Well-being, health and performance of young employees with intermediate education. *Career Dev. Int.* 14 671–699. 10.1108/13620430911005717

[B2] Akkermans J. Brenninkmeijer V. Huibers M. Blonk R. W. B. (2013a). Competencies for the contemporary career: development and preliminary validation of the career competencies questionnaire. *J. Career Dev.* 40 245–267. 10.1177/0894845312467501

[B3] Akkermans J. Schaufeli W. B. Brenninkmeijer V. Blonk R. W. B. (2013b). The role of career competencies in the job Demands — Resources model. *J. Vocat. Behav.* 83 356–366. 10.1016/j.jvb.2013.06.011

[B4] Akkermans J. Brenninkmeijer V. Schaufeli W. B. Blonk R. W. B. (2015a). It’s all about CareerSKILLS: effectiveness of a career development intervention for young employees. *Hum. Resour. Manag.* 54 533–551. 10.1002/hrm.21633

[B5] Akkermans J. Nykänen M. Vuori J. (2015b). “Practice makes Perfect? Antecedents and Consequences of an Adaptive School-to-Work Transition,” in *Sustainable Working Lives - Managing Work Transitions and Health throughout the Life Course*, eds Vuori J. Blonk R. W. B. Price R. (London: Springer Publishers), 65–86.

[B6] Akkermans J. Tims M. (2017). Crafting your career: how career competencies relate to career success via job crafting. *Appl. Psychol.* 66 168–195. 10.1111/apps.12082

[B7] Alarcon G. M. Lyons J. B. (2011). The relationship of engagement and job satisfaction in working samples. *J. Psychol.* 145 463–480. 10.1080/00223980.2011.584083 21902012

[B8] Bakker A. B. Demerouti E. (2007). The job demands-resources model: state of the art. *J. Manag. Psychol.* 22 309–328. 10.1108/02683940710733115

[B9] Bakker A. B. Demerouti E. (2014). *Job Demands–Resources Theory in Wellbeing.* Hoboken, NJ: John Wiley & Sons, Ltd.

[B10] Bakker A. B. Demerouti E. (2017). Job demands–resources theory: taking stock and looking forward. *J. Occup. Health Psychol.* 22 273–285. 10.1037/ocp0000056 27732008

[B11] Bakker A. B. Sanz Vergel A. I. Kuntze J. (2015). Student engagement and performance: a weekly diary study on the role of openness. *Motiv. Emot.* 39 49–62. 10.1007/s11031-014-9422-5

[B12] Beaton D. E. Bombardier C. Guillemin F. Ferraz M. B. (2000). Guidelines for the process of cross-cultural adaptation of self-report measures. *Spine* 25 3186–3191. 10.1097/00007632-200012150-00014 11124735

[B13] Boehm J. K. Lyubomirsky S. (2008). Does happiness promote career success? *J. Career Assess.* 16 101–116. 10.1177/1069072707308140

[B14] Boyd C. M. Bakker A. B. Pignata S. Winefield A. H. Gillespie N. Stough C. (2011). A longitudinal test of the job demands-resources model among australian university academics. *Appl. Psychol.* 60 112–140. 10.1111/j.1464-0597.2010.00429.x 27610093

[B15] Bresó E. Schaufeli W. B. Salanova M. (2011). Can a self-efficacy-based intervention decrease burnout, increase engagement, and enhance performance? A quasi-experimental study. *High. Educ.* 61 339–355. 10.1007/s10734-010-9334-6

[B16] Brislin R. W. (1970). Back-translation for cross-cultural research. *J. Cross Cult. Psychol.* 1 185–216. 10.1177/135910457000100301

[B17] Buyukgoze-Kavas A. Duffy R. D. Douglass R. P. (2015). Exploring links between career adaptability, work volition, and well-being among Turkish students. *J. Vocat. Behav.* 90 122–131. 10.1016/j.jvb.2015.08.006

[B18] Byrne B. M. (2012). *Structural equation modeling with Mplus: Basic concepts, applications, and programming.* New York, NY: Routledge.

[B19] Cardoso P. Silva J. R. Gonçalves M. M. Duarte M. E. (2014). Innovative moments and change in career construction counseling. *J. Vocat. Behav.* 84 11–20. 10.1016/j.jvb.2013.10.001

[B20] Chen F. F. (2007). Sensitivity of goodness of fit indexes to lack of measurement invariance. *Struct. Equ Modeling* 14 464–504. 10.1080/10705510701301834

[B21] Christian M. S. Garza A. S. Slaughter J. E. (2011). Work engagement: a quantitative review and test of its relations with task and contextual performance. *Pers. Psychol.* 64 89–136. 10.1111/j.1744-6570.2010.01203.x

[B22] Cox D. W. Krieshok T. S. Bjornsen A. L. Zumbo B. D. (2015). Occupational engagement scale–student: development and initial validation. *J. Career Assess.* 23 107–116. 10.1177/1069072714523090

[B23] De Vos A. De Clippeleer I. Dewilde T. (2009). Proactive career behaviours and career success during the early career. *J. Occup. Organ. Psychol.* 82 761–777. 10.1348/096317909X471013

[B24] De Vos A. Van Der Heijden B. I. J. M. Akkermans J. (2018). Sustainable careers: towards a conceptual model. *J. Vocat. Behav.* (in press) 10.1016/j.jvb.2018.06.011

[B25] Demerouti E. Bakker A. B. Nachreiner F. Schaufeli W. B. (2001). The job demands-resources model of burnout. *J. Appl. Psychol.* 86:499 10.1037/0021-9010.86.3.499 11419809

[B26] Diener E. Chan M. Y. (2011). Happy people live longer: subjective well-being contributes to health and longevity. *Appl. Psychol* 3 1–43. 10.1111/j.1758-0854.2010.01045.x

[B27] Diener E. Emmons R. A. Larsen R. J. Griffin S. (1985). The satisfaction with life scale. *J. Pers. Assess.* 49 71–75. 10.1207/s15327752jpa4901_13 16367493

[B28] Duffy R. D. Douglass R. P. Autin K. L. (2015). Career adaptability and academic satisfaction: examining work volition and self-efficacy as mediators. *J. Vocat. Behav.* 90 46–54. 10.1016/j.jvb.2015.07.007

[B29] Forrier A. Verbruggen M. De Cuyper N. (2015). Integrating different notions of employability in a dynamic chain: the relationship between job transitions, movement capital and perceived employability. *J. Vocat. Behav.* 89 56–64. 10.1016/j.jvb.2015.04.007

[B30] Hartung P. J. Taber B. J. (2008). Career construction and subjective well-being. *J. Career Assess.* 16 75–85. 10.1177/1069072707305772

[B31] Hirschi A. (2009). Career adaptability development in adolescence: multiple predictors and effect on sense of power and life satisfaction. *J. Vocat. Behav.* 74 145–155. 10.1016/j.jvb.2009.01.002

[B32] Hirschi A. (2012). The career resources model: an integrative framework for career counsellors. *Br. J. Guidance Couns.* 40 369–383. 10.1080/03069885.2012.700506

[B33] Hirschi A. (2014). Hope as a resource for self-directed career management: investigating mediating effects on proactive career behaviors and life and job satisfaction. *J. Happiness Stud.* 15 1495–1512. 10.1007/s10902-013-9488-x

[B34] Hirschi A. Herrmann A. Keller A. C. (2015). Career adaptivity, adaptability, and adapting: a conceptual and empirical investigation. *J. Vocat. Behav.* 87 1–10. 10.1016/j.jvb.2014.11.008

[B35] Hobfoll S. E. (2002). Social and psychological resources and adaptation. *Rev. Gen. Psychol.* 6 307–324. 10.1037/1089-2680.6.4.307

[B36] Howell A. J. (2009). Flourishing: achievement-related correlates of students’ well-being. *J. Posit. Psychol.* 4 1–13. 10.1080/17439760802043459

[B37] Infante E. Marín M. (2008). “Psychosocial profile of unsuccessful university student: Personality and motivational factors revisited,” in *Advances in Psychology Research*, ed. Columbus A. M. (Hauppauge, NY: NovaScience), 135–149.

[B38] Jackson D. Chapman E. (2012). Non-technical skill gaps in Australian business graduates. *Educ. Train.* 54 95–113. 10.1108/00400911211210224

[B39] Judge T. A. Van Vianen A. E. M. De Pater I. E. (2004). Emotional stability, core self-evaluations, and job outcomes: a review of the evidence and an agenda for future research. *Hum. Perform.* 17 325–346. 10.1207/s15327043hup17034

[B40] Kim K. R. Seo E. H. (2015). The relationship between procrastination and academic performance: a meta-analysis. *Pers. Individ. Differ.* 82 26–33. 10.1016/j.paid.2015.02.038

[B41] King Z. (2004). Career self-management: its nature, causes and consequences. *J. Vocat. Behav.* 65 112–133. 10.1016/S0001-8791(03)00052-6 23796465

[B42] Knight C. Patterson M. Dawson J. (2017). Building work engagement: a systematic review and meta-analysis investigating the effectiveness of work engagement interventions. *J. Organ. Behav.* 38 792–812. 10.1002/job.2167 28781428 PMC5516176

[B43] Koen J. Klehe U.-C. Van Vianen A. E. M. (2012). Training career adaptability to facilitate a successful school-to-work transition. *J. Vocat. Behav.* 81 395–408. 10.1016/j.jvb.2012.10.003

[B44] Kuijpers M. A. C. T. Schyns B. Scheerens J. (2006). Career competencies for career success. *Career Dev. Q.* 55 168–178. 10.1002/j.2161-0045.2006.tb00011.x

[B45] Lent R. W. Brown S. D. (2013). Social cognitive model of career self-management: toward a unifying view of adaptive career behavior across the life span. *J. Couns. Psychol.* 60 557–568. 10.1037/a0033446 23815631

[B46] Maggiori C. Johnston C. S. Krings F. Massoudi K. Rossier J. (2013). The role of career adaptability and work conditions on general and professional well-being. *J. Vocat. Behav.* 83 437–449. 10.1016/j.jvb.2013.07.001

[B47] Maggiori C. Rossier J. Savickas M. L. (2017). Career adapt-abilities scale–short form (CAAS-SF): construction and validation. *J. Career Assess.* 25 312–325. 10.1177/1069072714565856 27047406

[B48] Mäkikangas A. Kinnunen U. Feldt T. (2004). Self-esteem, dispositional optimism, and health: evidence from cross-lagged data on employees. *J. Res. Pers.* 38 556–575. 10.1016/j.jrp.2004.02.001

[B49] McIlveen P. Beccaria G. Burton L. J. (2013). Beyond conscientiousness: career optimism and satisfaction with academic major. *J. Vocat. Behav.* 83 229–236. 10.1016/j.jvb.2013.05.005

[B50] Mokgele K. R. Rothmann S. (2014). A structural model of student well-being. *South Afr. J. Psychol.* 44 514–527. 10.1177/0081246314541589

[B51] Muthén L. K. Muthén B. O. (2012). *MPlus User’s Guide.* Los Angeles, CA: Muthén & Muthén.

[B52] Nauta M. M. (2007). Assessing college students’ satisfaction with their academic majors. *J. Career Assess.* 15 446–462. 10.1177/1069072707305762

[B53] Negru-Subtirica O. Pop E. I. (2016). Longitudinal links between career adaptability and academic achievement in adolescence. *J. Vocat. Behav.* 93 163–170. 10.1016/j.jvb.2016.02.006

[B54] Ouweneel E. Le Blanc P. M. Schaufeli W. B. (2013). Do-it-yourself: an online positive psychology intervention to promote positive emotions, self-efficacy, and engagement at work. *Career Dev. Int.* 18 173–195. 10.1108/CDI-10-2012-0102

[B55] Pavot W. Diener E. (1993). Review of the satisfaction with life Scale. *Psychol. Assess.* 5 164–172. 10.1037/1040-3590.5.2.164

[B56] Podsakoff P. M. MacKenzie S. B. Lee J.-Y. Podsakoff N. P. (2003). Common method biases in behavioral research: a critical review of the literature and recommended remedies. *J. Appl. Psychol.* 88 879–903. 10.1037/0021-9010.88.5.879 14516251

[B57] Proctor C. L. Linley P. A. Maltby J. (2009). Youth life satisfaction: a review of the literature. *J. Happiness Stud.* 10 583–630. 10.1007/s10902-008-9110-9

[B58] Reis D. Hoppe A. Schröder A. (2015). Reciprocal relationships between resources, work and study engagement, and mental health: evidence for gain cycles. *Eur. J. Work Organ. Psychol.* 24 59–75. 10.1080/1359432X.2013.834891

[B59] Richardson M. Abraham C. Bond R. (2012). Psychological correlates of university students’ academic performance: a systematic review and meta-analysis. *Psychol. Bull.* 138 353. 10.1037/a0026838 22352812

[B60] Robbins S. B. Lauver K. Le H. Davis D. Langley R. Carlstrom A. (2004). Do psychosocial and study skill factors predict college outcomes? A meta-analysis. *Psychol. Bull.* 130 261–288. 10.1037/0033-2909.130.2.261 14979772

[B61] Robins T. G. Roberts R. M. Sarris A. (2015). Burnout and engagement in health profession students: the relationships between study demands, study resources and personal resources. *Austr. J. Organ. Psychol.* 8:e1 10.1017/orp.2014.7

[B62] Robotham D. Julian C. (2006). Stress and the higher education student: a critical review of the literature. *J. Further High. Educ.* 30 107–117. 10.1080/03098770600617513

[B63] Rottinghaus P. J. Day S. X. Borgen F. H. (2005). The career futures inventory: a measure of career-related adaptability and optimism. *J. Career Assess.* 13 3–24. 10.1177/1069072704270271

[B64] Rudolph C. W. Lavigne K. N. Zacher H. (2017). Career adaptability: a meta-analysis of relationships with measures of adaptivity, adapting responses, and adaptation results. *J. Vocat. Behav.* 98(Suppl. C), 17–34. 10.1016/j.jvb.2016.09.002

[B65] Ryan P. (2001). The school-to-work transition: a cross-national perspective. *J. Econ. Literat.* 39 34–92.

[B66] Salanova M. Schaufeli W. Martínez I. Bresó E. (2010). How obstacles and facilitators predict academic performance: the mediating role of study burnout and engagement. *Anxiety Stress Coping* 23 53–70. 10.1080/10615800802609965 19326271

[B67] Salmela-Aro K. Kunttu K. (2010). Study burnout and engagement in higher education. *Unterrichtswissenschaft* 38 318–333.

[B68] Salmela-Aro K. Upadyaya K. (2014). School burnout and engagement in the context of demands–resources model. *Br. J. Educ. Psychol.* 84 137–151. 10.1111/bjep.12018 24547758

[B69] Savickas M. L. (1997). Career adaptability: an integrative construct for life-span, life-space theory. *Career Dev. Q.* 45 247–259. 10.1002/j.2161-0045.1997.tb00469.x

[B70] Savickas M. L. (2005). “The theory and practice of career construction,” in *Career development and counseling*, eds Brown S. D. Lent R. W. (Hoboken, NJ: John Wiley & Sons, Inc.), 42–70.

[B71] Savickas M. L. Nota L. Rossier J. Dauwalder J.-P. Duarte M. E. Guichard J. (2009). Life designing: a paradigm for career construction in the 21st century. *J. Vocat. Behav.* 75 239–250. 10.1016/j.jvb.2009.04.004

[B72] Savickas M. L. Porfeli E. J. (2012). Career adapt-abilities scale: construction, reliability, and measurement equivalence across 13 countries. *J. Vocat. Behav.* 80 661–673. 10.1016/j.jvb.2012.01.011

[B73] Schaufeli W. B. Bakker A. B. Salanova M. (2006). The measurement of work engagement with a short questionnaire: a cross-national study. *Educ. Psychol. Meas.* 66 701–716. 10.1177/0013164405282471

[B74] Schoon I. McCulloch A. Joshi H. E. Wiggins R. D. Bynner J. (2001). Transitions from school to work in a changing social context. *Young* 9 4–22. 10.1177/110330880100900102 16775603

[B75] Sovet L. Park M. S.-A. Jung S. (2014). Validation and psychometric properties of academic major satisfaction scale among Korean college students. *Soc. Indicat. Res.* 119 1121–1131. 10.1007/s11205-013-0537-y

[B76] Strenze T. (2007). Intelligence and socioeconomic success: a meta-analytic review of longitudinal research. *Intelligence* 35 401–426. 10.1016/j.intell.2006.09.004

[B77] Sullivan S. E. Baruch Y. (2009). Advances in career theory and research: a critical review and agenda for future exploration. *J. Manag.* 35 1542–1571. 10.1177/0149206309350082

[B78] Urbanaviciute I. Pociute B. Kairys A. Liniauskaite A. (2016). Perceived career barriers and vocational outcomes among university undergraduates: exploring mediation and moderation effects. *J. Vocat. Behav.* 92 12–21. 10.1016/j.jvb.2015.11.001

[B79] Vandenberg R. J. Lance C. E. (2000). A review and synthesis of the measurement invariance literature: suggestions, practices, and recommendations for organizational research. *Organ. Res. Methods* 3 4–70. 10.1177/109442810031002

[B80] Vermeulen L. Schmidt H. G. (2008). Learning environment, learning process, academic outcomes and career success of university graduates. *Stud. High. Educ.* 33 431–451. 10.1080/03075070802211810

[B81] Whitelock D. Thorpe M. Galley R. (2015). Student workload: a case study of its significance, evaluation and management at the Open University. *Distance Educ.* 36 161–176. 10.1080/01587919.2015.1055059

[B82] Xanthopoulou D. Bakker A. B. Demerouti E. Schaufeli W. B. (2007). The role of personal resources in the job demands-resources model. *Int. J. Stress Manag.* 14 121–141. 10.1037/1072-5245.14.2.121

[B83] Xanthopoulou D. Bakker A. B. Demerouti E. Schaufeli W. B. (2009). Reciprocal relationships between job resources, personal resources, and work engagement. *J. Vocat. Behav.* 74 235–244. 10.1016/j.jvb.2008.11.003

